# E2F transcription factor 2-activated DLEU2 contributes to prostate tumorigenesis by upregulating serum and glucocorticoid-induced protein kinase 1

**DOI:** 10.1038/s41419-022-04525-1

**Published:** 2022-01-24

**Authors:** Peizhang Li, Huan Xu, Liu Yang, Ming Zhan, Yuanping Shi, Caoxu Zhang, Dajun Gao, Meng Gu, Yanbo Chen, Zhong Wang

**Affiliations:** 1 Department of Urology, Shanghai Ninth People’s Hospital Affiliated to Shanghai Jiaotong University School of Medicine Shanghai, Shanghai, China; 2 The Core Laboratory in Medical Center of Clinical Research, Department of Molecular Diagnostics and Endocrinology, Shanghai Ninth People’s Hospital, Shanghai Jiaotong University School of Medicine, Shanghai, China

**Keywords:** Oncogenes, Tumour biomarkers, Prostate cancer

## Abstract

Long noncoding RNAs (lncRNAs) participate in biological processes in multiple types of tumors. However, the regulatory patterns of lncRNAs in prostate cancer remain largely unclear. Here, we evaluated the expression and roles of the lncRNA DLEU2 in prostate cancer. Our results showed that DLEU2 was upregulated in advanced prostate cancer tissues. Patients with prostate cancer displaying high expression of DLEU2 had a poor prognosis. Moreover, we demonstrated that overexpression of DLEU2 facilitated the proliferation, migration, and invasion of prostate cancer in vitro. Mechanistically, DLEU2 promoted serum and glucocorticoid-induced protein kinase 1 (SGK1) expression by acting as an miR-582-5p sponge, and the transcription of DLEU2 was activated by the dysregulation of E2F transcription factor 2 (E2F2) expression in prostate cancer. Furthermore, knockdown of DLEU2 attenuated prostate cancer tumorigenesis in vivo. Notably, these findings suggested that E2F2-activated DLEU2 may function as a competing endogenous RNA to facilitate prostate cancer progression by targeting the miR-582-5p/SGK1 axis.

## Introduction

Prostate cancer is the leading cause of cancer-related death in men in the United States of America [1, 2]. Androgen-deprivation therapy (ADT) is the first-line therapy for patients diagnosed with prostate cancer and has improved overall survival (OS) in men with metastatic prostate cancer [3]; however, most patients develop resistance to ADT after 12–36 months of treatment [4, 5]. The underlying mechanisms of prostate carcinogenesis are still poorly understood [6]. Therefore, further studies of the genetic and epigenetic molecular mechanisms of prostate cancer are critical for disease management.

Long noncoding RNAs (lncRNAs) are functional transcripts greater than 200 nucleotides in length without protein-coding ability. LncRNAs can bind to DNA, RNA, and protein to form complexes, acting as signals, decoys, guides, and scaffolds [7, 8]. Additionally, lncRNAs promote or suppress transcription and stabilize or destabilize mRNAs or proteins [9]. Moreover, lncRNAs play important roles in various diseases, particularly cancer [10–12]. For example, the lncRNA MILIP is involved in cell proliferation, division, and tumorigenicity by inducing p53 degradation. MILIP also suppresses tripartite motif family-like 2-mediated p53 SUMOylation and promotes p53 polyubiquitination [13]. PCAT1 stimulates prostate cancer progression by activating AKT and nuclear factor (NF)-κB signaling [14]. Furthermore, the lncRNA DLEU2 is overexpressed in esophageal cancer tissue and is related to poor prognosis in esophageal cancer patients. DLEU2 also promotes the proliferation, migration, and invasion of esophageal cancer cells via the miR-30e-5p/E2F transcription factor 7 axis [15]. Other studies have shown that DLEU2 has oncogenic roles in non-small cell lung cancer and hepatocellular carcinoma [16, 17]. However, the functional roles and mechanisms of DLEU2 in prostate cancer progression are largely unknown.

Serum and glucocorticoid-induced protein kinase 1 (SGK1) is a serine/threonine kinase that plays important roles in regulating multiple ion channels, membrane transporters, and cellular enzymes [18–20]. SGK1 is also involved in various physiological processes, such as memory consolidation, reproductive process, and cell growth [18, 21, 22]. Notably, SGK1 has been shown to be dysregulated in multiple cancers, including prostate cancer, and to participate in the regulation of tumor development [23–27]. However, the underlying mechanisms of dysregulated SGK1 expression in tumors are largely unknown.

E2F transcription factor 2 (E2F2) is widely expressed in many tissues and organs and activates transcription to modulate cellular proliferation, differentiation, cell cycle, and DNA repair [28–30]. E2F2 is highly expressed in multiple types of tumors [31]. For example, E2F2 is required for hepatocellular carcinoma development, and E2F2 deletion confers protection against hepatocarcinogenesis by preventing lipid storage [32]. Similarly, E2F2 is upregulated and promotes the malignant activities of cancer cells in pancreatic cancer and osteosarcoma. However, its novel regulatory mechanism in prostate cancer needs further investigation.

Accordingly, in this study, we attempted to illustrate the contributions and mechanisms of lncRNA DLEU2 in prostate cancer. We demonstrated that high expression of DLEU2 facilitated prostate cancer progression, including proliferation, colony formation, migration, and invasion. We further indicated that DLEU2 could improve SGK1 expression via competing interacting with miR-582-5p. In addition, we found that E2F2 regulated DLEU2 expression by directly binding to DLEU2 promoter. Collectively, our findings provided insights into the oncogenic roles of DLEU2 in prostate cancer progression.

## Materials and methods

### Cell lines and culture

The human prostate cancer cell lines PC-3 and DU145 were purchased from the national collection of authenticated cell culture at the Chinese Academy of Sciences (Shanghai, China) and were validated by short tandem repeat DNA profiling analysis. PC-3 and DU145 cells were cultured in MEM medium (Gibco; A4192201) with 10% fetal bovine serum (FBS; Gibco).

### Plasmids, short hairpin RNA (shRNA), microRNA (miRNA) mimics, and inhibitor construction

The DLEU2 and E2F2 overexpression vectors pcDNA3.1-DLEU2 and pcDNA3.1-E2F2 were constructed by Miaolingbio (Wuhan, China). pMIRGLO-DLEU2 and pMIRGLO-SGK1 were purchased from Miaolingbio. ShRNAs targeting DLEU2 and control shRNA were obtained from Genepharma (Shanghai, China). Mimics, miR-582-5p inhibitor, and miR-582-5p mimics were also synthesized by Genepharma.

### Total RNA extraction and real-time quantitative polymerase chain reaction (qPCR)

Total RNA was isolated from prostate cancer cells using TRIzol reagent (Invitrogen, USA) according to the manufacturer’s instructions. cDNA was synthesized by random primers and miRNA was reverse transcribed using miRNA-specific stem-loop primers. Real-time qPCR was performed on a Bio-Rad CFX96TM Real-Time PCR System (Bio-Rad Laboratories, Hercules, CA, USA). qPCR primers were purchased from BioSune (Shanghai, China). Relative RNA expression was analyzed using the 2^-ΔΔCt^ method. The primers are shown in Supplementary Table S1.

### Transfection

Plasmids, shRNAs, miRNA mimics, and miRNA inhibitors were transfected into cancer cells using Lipofectamine 2000 (Invitrogen) according to the manufacturer’s instructions. To obtain stable cell lines showing low expression of DLEU2, we transfected the negative control vector or low-expression DLEU2 vector into PC-3 and DU145 cells and then selected transfected cells using puromycin (Sangon Biotech, China) for 2–3 weeks until DLEU2 was stably expressed at low levels in the cells.

### Cell proliferation assay

Cell proliferation was measured using Cell Counting Kit-8 (CCK-8) and 5-ethynyl-2-deoxyuridine (EdU) assays. For CCK-8 assays, cells were seeded into 96-well plates at a density of 3000 cells/well and incubated for 1 h in 10% CCK-8 medium at 0, 24, 48, and 72 h after seeding. The absorbance was detected at an optical density of 450 nm with a spectrophotometer. For EdU assays, cells were seeded into 96-well plates and were incubated with 10 μM EdU for 1 h at 37 °C. After fixation with 4% paraformaldehyde (PFA) and permeabilization in phosphate-buffered saline (PBS) with 0.3% Triton X-100, cells were exposed to 100 μL Click-iT kfluor488 mixture for 30 min and were incubated with DAPI for 15 min. The EdU-positive cells/DAPI-positive cells were measured to determine the proliferation rate.

### Colony formation assays

The treated cells were incubated in 6-well plates at a density of 200 cells/well and cultured for 2 weeks. Half of the medium was changed every 3 days. The cells were then fixed with 4% PFA and subsequently examined with crystal violet staining.

### Transwell assays

For transwell migration assays, 20000 cells were counted, resuspended in 200 μL MEM medium without FBS, and seeded in the upper chambers of transwell inserts (Corning, USA). The lower chambers were loaded with MEM medium containing 20% FBS. For transwell invasion assays, 30000 treated cells were resuspended in 200 μL MEM medium without FBS and seeded in the upper chambers of transwell inserts coated with Matrigel (BD Biosciences, USA). The cells were incubated for 18 h for migration assays or 24 h for invasion assays. Then, the cells on the lower surface were fixed and stained with crystal violet staining solution. Images were captured with a microscope, and the number of cells was counted using ImageJ.

### RNA fluorescence in situ hybridization (FISH)

Cy3-labeled DLEU2 probes were designed and synthesized by Genepharma. The sequence of the probes was as follows: DLEU2-Cy3 for FISH, AGTGAGGCTGT + TCTCCAGAAT + TGGT. The cells were fixed using 4% PFA for 20 min and were then incubated with prehybridization buffer. Then, hybridization was conducted at 55 °C for 2 h. After staining with DAPI, cells were incubated with DLEU2 probes using a FISH kit according to the manufacturer’s instructions. Confocal microscopy was used to capture the images.

### Western blotting

Total proteins were obtained using RIPA lysis buffer (Sangon Biotech) containing 1% protease inhibitor cocktail (Bimake). The proteins were separated by sodium dodecyl sulfate (SDS) polyacrylamide gel electrophoresis and transferred to VEDF membranes (Merck Millipore, Germany). After blocking with 5% bovine serum albumin, the membranes were incubated with primary antibodies against β-actin (cat. no. 4970 s; Cell Signaling Technology, Danvers, MA, USA) or SGK1 (cat. no. ab32374; Abcam, Cambridge, MA, USA) overnight at 4 °C. Then, the membranes were washed with TBST buffer and incubated with horseradish peroxidase-conjugated secondary antibodies for 1 h at room temperature. The images were analyzed using ImageJ.

### Luciferase reporter assay

Prostate cancer cells were seeded into 24-well plates at a density of 30000 cells/well. miR-582-5p mimics or negative control were cotransfected with luciferase reporter plasmids into the cells using Lipofectamine 2000 (Invitrogen). After 48 h, the cells were digested with 0.25% pancreatin and collected. The luciferase activities of the cells were measured using a Dual Luciferase Assay Kit (Promega, Madison, WI, USA) according to the manufacturer’s protocol.

### Chromatin immunoprecipitation (ChIP) assay

In total, 1 × 10^7^ cells were crosslinked in 1% formaldehyde for 10 min at 37 °C. After washing with PBS, the cells were resuspended in 300 μL lysis buffer, and the DNA was sheared to obtain 200–1000 bp fragments using sonication. Sonicated chromatin was diluted to a final concentration of 0.1% SDS. Then, the aliquots were incubated with anti-Flag antibodies or isotype control IgG for 2 h. The immunoprecipitated DNA was retrieved from Protein A/G Magnetic beads (Bimake) with 1% SDS and a 1.1 M NaHCO3 solution at 65 °C for 6 h. Subsequently, the DNA was purified using a PCR Purification Kit (Cell Signaling Technology) and quantified using real-time qPCR.

### In vivo experiments

A subcutaneous xenograft mouse model was established to evaluate the tumor formation ability of control and DLEU2-knockdown PC-3 cells. All animal experiments were carried out under specific pathogen-free conditions at the animal care facility of the Experimental Animal Center of National Dong-Hua University. Four-week-old male BALB/c nude mice (weighing 20–25 g) were obtained from Shanghai Jie Si Jie Laboratory Animal Ltd. For the subcutaneous xenograft experiment, 1 × 10^6^ control or DLEU2-knockdown PC-3 cells were resuspended in 100 μL of 1× PBS and injected subcutaneously into the flanks of BALB/c nude mice. The lengths and widths of tumors were measured every 5 days, and the tumor volume was calculated according to the following formula: volume = 0.5 × length × width^2^. Thirty-five days later, all mice were anesthetized and sacrificed, and the tumors were then resected and collected for immunohistochemistry (IHC) and FISH analysis.

### Bioinformatics analysis

Gene Expression Profiling Interactive Analysis (GEPIA; http://gepia.cancer-pku.cn/) was used to obtain the list of genes affected by the lncRNA DLEU2. The target miRNAs of DLEU2 and SGK1 were predicted using ENCORI (http://starbase.sysu.edu.cn/) [33]. The binding sites for E2F2 in the DLEU2 promoter region were predicted using Jaspar (http://jaspar.genereg.net/).

### Statistical analysis

The statistical analyses were performed using SPSS (22.0). Data are presented as means ± standard deviations from triplicates. Results with *P* values less than 0.05 were considered significant. T-tests were used to determine differences between two groups, and one-way analysis of variance was used to determine differences between multiple groups.

## Results

### DLEU2 was highly expressed in advanced prostate cancer and was associated with a poor prognosis

**Fig. 1:**
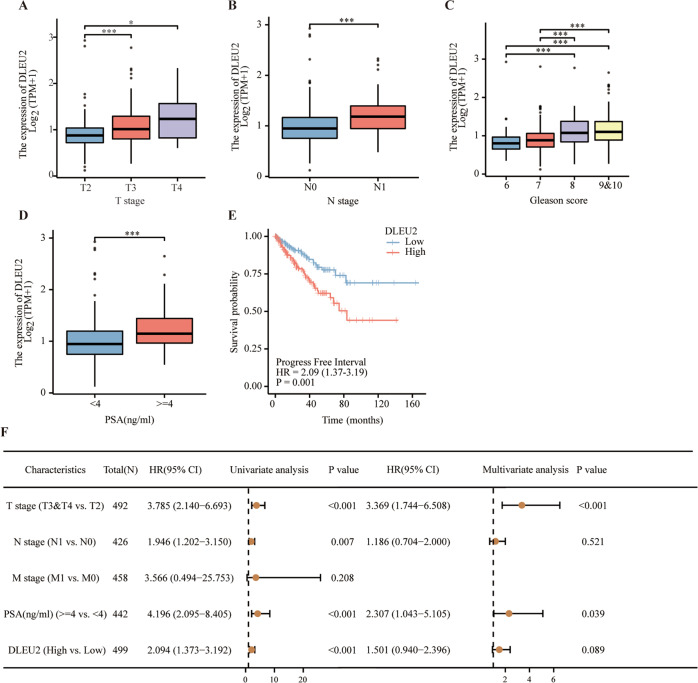
DLEU2 was highly expressed in advanced prostate cancer and was associated with a poor prognosis. **A** The correlation between T stage and DLEU2 expression in prostate cancer. **B** The correlation between N stage and DLEU2 expression. **C** The correlation between Gleason scores and DLEU2 expression. **D** The correlation between PSA levels and DLEU2 expression. **E** Kaplan Meier curves showed the correlation between DLEU2 expression and PFI in patients with prostate cancer according to TCGA database. **F** Multivariable analysis of hazard ratios for PFI in prostate cancer was performed by using logistic regression model. Data were indicated as mean ± SD, ns *P* ≥ 0.05, *P < 0.05, **P < 0.01, ***P < 0.001.

First, we investigated the expression of DLEU2 in prostate tumor tissues from The Cancer Genome Atlas (TCGA) database. DLEU2 expression was higher in T III/IV stage samples than in T II stage samples (Fig. 1A), and high DLEU2 expression was associated with more advanced N stage, higher Gleason score, and prostate-specific antigen (PSA) level (Fig. 1B–D). DLEU2 expression and other clinical features in patients with prostate cancer were analyzed (Table 1). Univariate analysis using logistic regression showed that higher DLEU2 expression was an independent variable and was correlated with poor prognosis (Table 2). Kaplan–Meier survival curves showed that patients with higher DLEU2 expression had lower survival rates (Fig. 1E). Consistent with this, high DLEU2 expression was independently associated with a poor progression-free interval according to univariate and multivariate Cox regression analysis (PFI; *P* < 0.1; Fig. 1F). Taken together, these results revealed that DLEU2 was highly expressed in advanced prostate cancer and was correlated with poor prognosis.

**Table 1:** Patients’ information in the TCGA.

Characteristic	Low expression of DLEU2	High expression of DLEU2	*p*
*n*	249	250	
T stage, *n* (%)			<0.001
T2	118 (24%)	71 (14.4%)	
T3	123 (25%)	169 (34.3%)	
T4	4 (0.8%)	7 (1.4%)	
N stage, *n* (%)			<0.001
N0	178 (41.8%)	169 (39.7%)	
N1	21 (4.9%)	58 (13.6%)	
M stage, *n* (%)			0.248
M0	227 (49.6%)	228 (49.8%)	
M1	0 (0%)	3 (0.7%)	
Gleason score, *n* (%)			<0.001
6	33 (6.6%)	13 (2.6%)	
7	146 (29.3%)	101 (20.2%)	
8	23 (4.6%)	41 (8.2%)	
9	45 (9%)	93 (18.6%)	
10	2 (0.4%)	2 (0.4%)	
Residual tumor, n (%)			0.270
R0	166 (35.5%)	149 (31.8%)	
R1	67 (14.3%)	81 (17.3%)	
R2	3 (0.6%)	2 (0.4%)	
PFI event, *n* (%)			0.005
Alive	215 (43.1%)	190 (38.1%)	
Dead	34 (6.8%)	60 (12%)	
Age, *n* (%)			0.013
<=60	126 (25.3%)	98 (19.6%)	
>60	123 (24.6%)	152 (30.5%)

**Table 2:** DLEU2 expression associated with clinical pathological variables (logistic regression).

Characteristics	Total (*N*)	Odds Ratio (OR)	*P* value
T stage (T3&T4 vs. T2)	492	2.303 (1.591–3.353)	<0.001
N stage (N1 vs. N0)	426	2.909 (1.717–5.097)	<0.001
Primary therapy outcome (CR vs. PD&SD&PR)	438	0.532 (0.333–0.841)	0.007
Residual tumor (R1&R2 vs. R0)	468	1.321 (0.897–1.949)	0.159
PSA (ng/ml) (>=4 vs. <4)	442	3.071 (1.329–7.971)	0.013
Gleason score (9&10 vs. 6&7&8)	499	2.634 (1.761–3.983)	<0.001

### DLEU2 regulated proliferation, migration, and invasion in prostate cancer cells

**Fig. 2:**
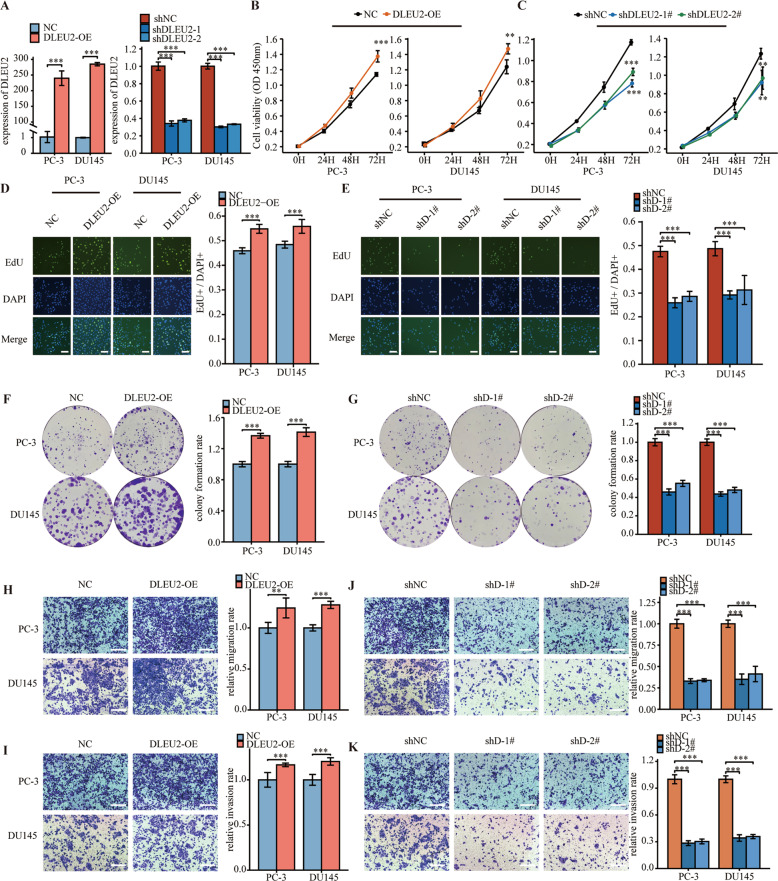
DLEU2 regulated proliferation, migration, and invasion in prostate cancer cells. **A** qPCR analysis showing the overexpression and knockdown efficiency of DLEU2. **B**, **C** Analysis of cell viability in DLEU2 overexpression and knockdown prostate cancer cells using CCK-8 kit. **D**, **E** Result of EdU assay showing the cell proliferation in DLEU2 overexpression and knockdown prostate cancer cells (scale bar: 50 μm). **F**, **G** Colony formation assay was performed to determine the proliferation of PC-3 and DU145 cells harboring the different vectors indicated. **H**, **I** Transwell assays were performed to determine the migration and invasion capacity of PC-3 and DU145 cells transfecting with DLEU2 (scale bar: 50 μm). **J**, **K** Transwell assays were performed to determine the migration and invasion capacity of PC-3 and DU145 cells stably expressing shRNAs targeting DLEU2 (scale bar: 50μm). Data were indicated as mean ± SD, ns *P* ≥ 0.05, *P < 0.05, **P < 0.01, ***P < 0.001.

To further explore the pathological roles of DLEU2 in prostate cancer, we first established DLEU2 overexpression and knockdown systems using pcDNA3.1-DLEU2 and sh-DLEU2 in PC-3 and DU145 cells. DLEU2 was dramatically upregulated or downregulated after transfection with pcDNA3.1-DLEU2 or sh-DLEU2, respectively (Fig. 2A), indicating that the systems were successfully established. DLEU2 overexpression significantly promoted PC-3 and DU145 cell growth (Fig. 2B). However, DLEU2 knockdown significantly inhibited PC-3 and DU145 cell growth (Fig. 2C). Similar results were obtained for proliferation analysis using EdU assays (Fig. 2D, E). Moreover, colony formation assays showed that colony numbers were higher in the DLEU2-overexpression group than in the negative control group (Fig. 2F) and were lower in the DLEU2-knockdown group than in the control group (Fig. 2G). Notably, DLEU2 overexpression strongly promoted the migration and invasion of prostate cancer cells (Fig. 2H, I), whereas DLEU2 knockdown significantly inhibited cell migration and invasion (Fig. 2J, K). Overall, these data confirmed that DLEU2 promoted the proliferation, migration, and invasion of prostate cancer cells in vitro.

### DLEU2 expression was positively correlated with SGK1 expression

**Fig. 3:**
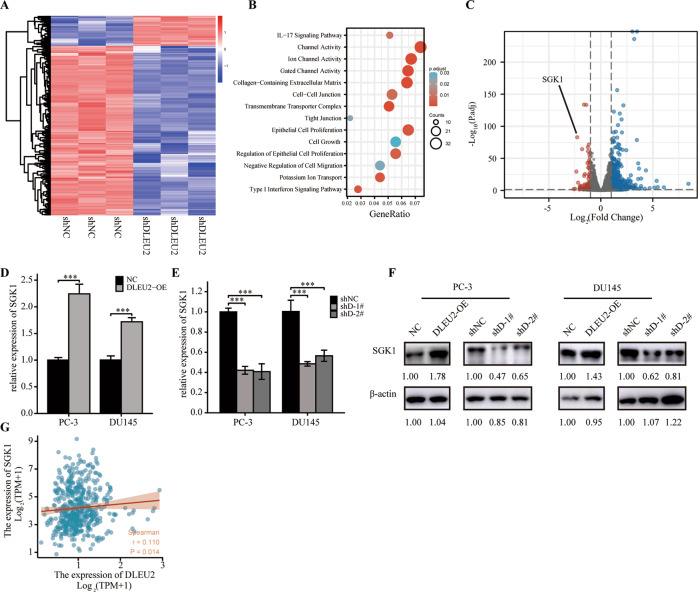
DLEU2 expression was positively correlated with SGK1 expression. **A** Heatmap illustrating the differentially expressed genes (DEGs) in PC-3 cells between shNC group and DLEU2 knockdown group. **B** GO and KEGG enrichment analysis of DEGs. **C** Volcano map showing the DEGs in PC-3 cells between shNC group and shDLEU2 group. **D**, **E** RT-qPCR result showing the expression of SGK1 PC-3 and DU145 cells. **F** Western blotting analysis of SGK1 expression in PC-3 and DU145 cells. **G** Correlation between SGK1 expression and DLEU2 expression in TCGA database using spearman analysis. Data were indicated as mean ± SD, ns *P* ≥ 0.05, *P < 0.05, **P < 0.01, ***P < 0.001.

To elucidate the molecular mechanisms of DLEU2 in prostate cancer progression, we performed mRNA-seq on PC-3 cells transfected with sh-NC or sh-DLEU2. Sequencing revealed 570 significantly differentially expressed genes, of which 484 were upregulated and 86 were downregulated (Fig. 3A). Gene Ontology and Kyoto Encyclopedia of Genes and Genomes analyses indicated that differentially expressed genes were associated with the cell cycle, cell proliferation, cell migration, and ion channel activity (Fig. 3B). Of these genes, SGK1 showed the greatest downregulation in DLEU2-knockdown cells (Fig. 3B). SGK1 encodes a serine/threonine-protein kinase involved in cellular stress responses and tumorigenesis. Therefore, we chose SGK1 for subsequent analyses.

RT-qPCR and western blotting indicated that SGK1 was distinctly upregulated in DLEU2-overexpressing prostate cancer cells (Fig. 3D) and downregulated in DLEU2-knockdown cells (Fig. 3E, F). These results demonstrated that DLEU2 promoted SGK1 expression. Moreover, SGK1 expression was positively correlated with DLEU2 expression (*r* = 0.110, *P* = 0.014; Fig. 3G) according to TCGA database.

### DLEU2 competed with SGK1 for interaction with miR-582-5p

**Fig. 4:**
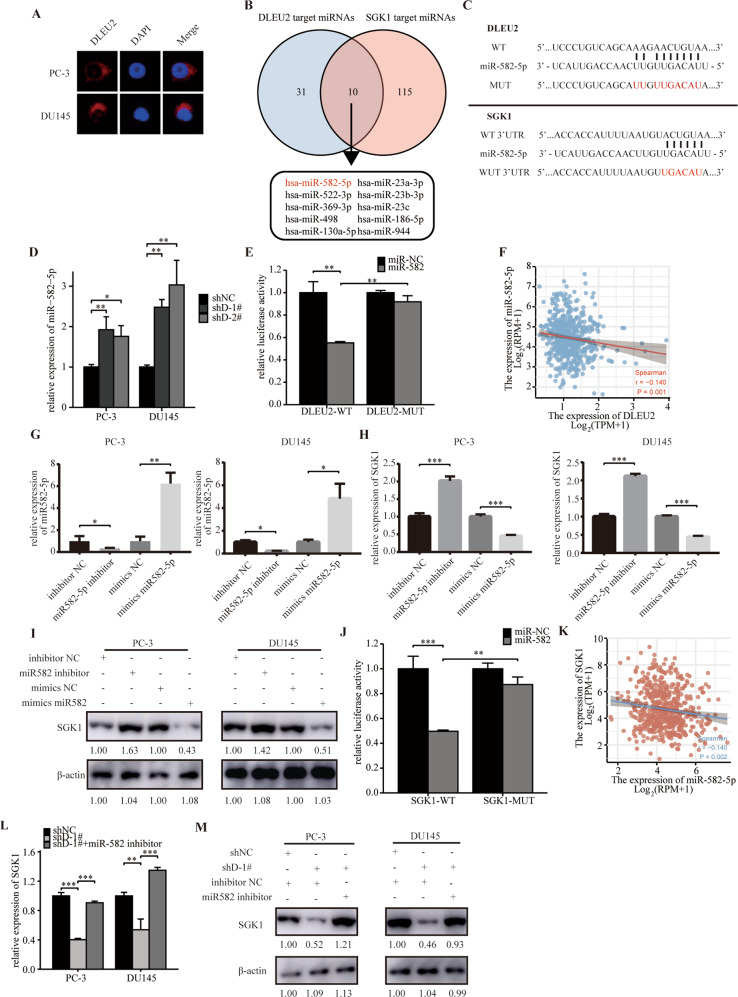
DLEU2 competed with SGK1 for interaction with miR-582-5p. **A** FISH analysis of subcellular localization of DLEU2 in PC-3 and DU145 cells. **B** Venn diagrams showing the miRNAs targeting DLEU2 and SGK1. **C** A schematic drawing showing the possible binding sites of miR-582-5p within DLEU2 and the SGK1 3′-UTR and the corresponding site-specific mutations. **D** Relative expression of miR-582-5p in PC-3 and DU145 cells stably knockdown DLEU2 using RT-qPCR. **E** Relative reporter gene activity of vector containing DLEU2 in 293 T cells co-transfected with miR-582-5p mimics. **F** Correlation between miR-582-5p expression and DLEU2 expression in TCGA database using spearman analysis. **G** Quantitative analysis of RNA levels of miR-582-5p in prostate cancer cells transfected with miR-582-5p inhibitor or miR-582-5p mimics. **H** Quantitative analysis of RNA levels of SGK1 in prostate cancer cells transfected with miR-582-5p inhibitor or miR-582-5p mimics. **I** Quantitative analysis of protein levels of SGK1 in prostate cancer cells transfected with miR-582-5p inhibitor or miR-582-5p mimics. **J** Relative luciferase reporter assays in 293 T cell lines co-transfected with vector containing the SGK1 3’UTR and miR-582-5p mimics. **K** Correlation between SGK1 expression and miR-582-5p expression in TCGA database using spearman analysis. **L**, **M** Quantitative analysis of SGK1 expression in prostate cancer cells co-transfected with shDLEU2 and miR582-5p inhibitor. Data were indicated as mean ± SD, ns *P* ≥ 0.05, *P < 0.05, **P < 0.01, ***P < 0.001.

To further determine the regulatory mechanism between DLEU2 and SGK1 in prostate cancer, we performed FISH to test subcellular location of DLEU2 in prostate cancer cells. Importantly, DLEU2 was mainly distributed in the cytoplasm of prostate cancer cells (Fig. 4A). In addition, we searched for NONCODE to assess the ability of DLEU2 to encode short peptides and the result showed that DLEU2 had no ability to encode short peptides (Fig. S1). LncRNAs in the cytoplasm can act as miRNA sponges to regulate mRNA expression [34]. Therefore, we hypothesized that DLEU2 may regulate SGK1 expression by competing for interactions with microRNAs. From ENCORI analysis, we found 41 miRNAs targeting DLEU2 and 125 miRNAs targeting SGK1 (Fig. 4B). Furthermore, among miRNAs targeting both DLEU2 and SGK1, miR-582-5p was found to be related to prostate cancer prognosis and clinicopathologic features (Fig. S2, Table S2); the potential binding sites of the two genes are shown in Fig. 4C. Therefore, miR-582-5p was selected for further studies.

Using PC-3 and DU145 cells, we found that miR-582-5p expression was upregulated following sh-DLEU2 transfection (Fig. 4D). Furthermore, in dual luciferase reporter assays, miR-582-5p decreased the luciferase activity of DLEU2-wild-type but not DLEU2-mutant (Fig. 4E). Spearman correlation analysis according to TCGA database showed a significant correlation between miR-582-5p and DLEU2 expression (Fig. 4F). Transfection with miR-582-5p inhibitor and mimic decreased and increased miR-582-5p expression, respectively (Fig. 4G), indicating that the knockdown and overexpression systems were established successfully. Further analyses in these knockdown and overexpression systems showed that SGK1 expression was negatively related to miR-582 expression (Fig. 4H, I), and dual-luciferase reporter assays confirmed that the 3′ untranslated region (UTR) of SGK1 was a direct target of miR-582-5p (Fig. 4J). Consistent with these findings, Spearman correlation analysis according to TCGA database showed a negative correlation between SGK1 and miR-582-5p expression (Fig. 4K). Additionally, mRNA and protein expression of SGK1 was greatly down-regulated by knockdown of DLEU2, while the effect was partially rescued by miR-582-5p inhibitor (Fig. 4L, M). In order to verify our hypothesis, we constructed a DLEU overexpression mutation vector similar to the luciferase plasmid, and the result suggested that transfection of wild vectors can increase the expression of SGK1, while transfection of mutant vectors will completely restore regulation (Fig. S3). Overall, these data suggested that DLEU2 regulated SGK1 expression by competing for interaction with miR-582-5p.

### DLEU2 effected prostate cancer progression via the miR-582-5p/SGK1 axis

**Fig. 5:**
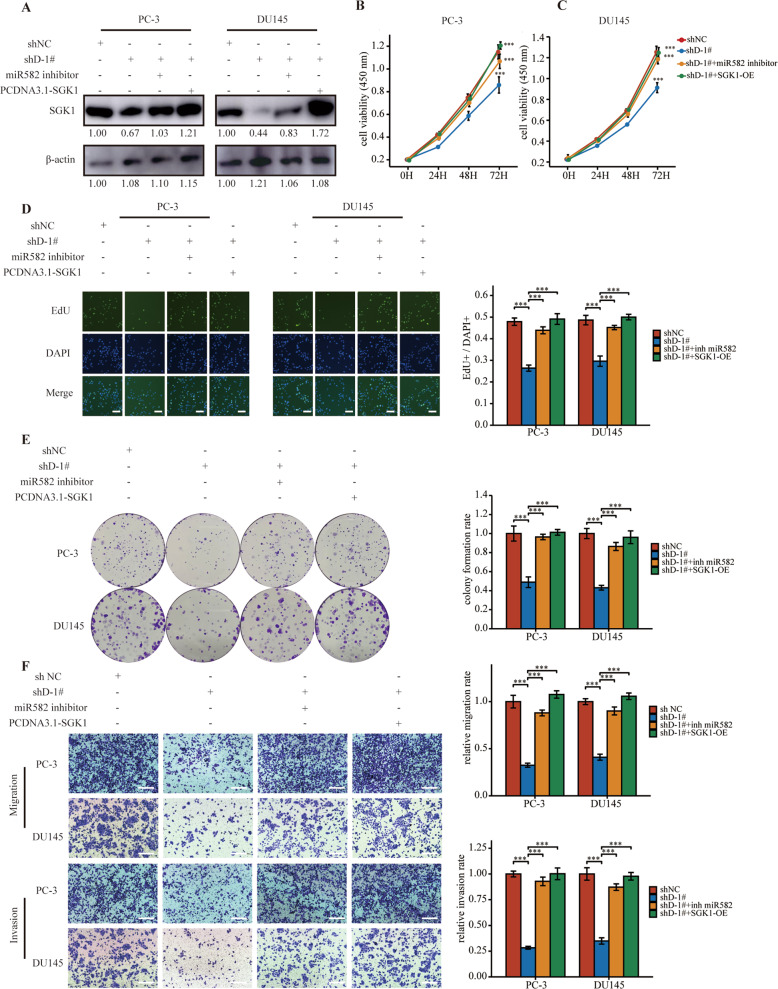
DLEU2 effected prostate cancer progression via the miR-582-5p/SGK1 axis. **A** Western blot analysis of SGK1 expression in DLEU2 knockdown PC-3, DU145 cells transfected with miR582-5p inhibitor or SGK1 overexpression vector. **B**, **C** The cell viability of PC-3 and DU145 cells as described above. **D** Result of EdU assay showing the cell proliferation in PC-3 and DU145 cells as described above (scale bar: 50 μm). **E** The colony formation analysis of PC-3 and DU145 cells as described above. **F** The migration and invasion analysis of prostate cancer cells as described above (scale bar: 50 μm). Data were indicated as mean ± SD, ns *P* ≥ 0.05, *P < 0.05, **P < 0.01, ***P < 0.001.

We then aimed to further clarify the roles of the DLEU2/miR-582-5p/SGK1 axis in prostate carcinogenesis by cotransfection with sh-DLEU2 and miR-582-5p inhibitor or PCDNA3.1-SGK1 in PC-3 cells and DU145 cells (Fig. 5A). Importantly, DLEU2 knockdown significantly inhibited the growth, proliferation, and colony formation of prostate cancer cells, and these effects were partially rescued by transfection with miR-582-5p inhibitor or SGK1 (Fig. 5B–E). Moreover, transwell migration and invasion assays demonstrated that DLEU2 knockdown blocked cell migration and invasion, and these effects were reversed by transfection with miR-582-5p inhibitor or overexpression of SGK1 (Fig. 5F). Taken together, these results demonstrated that DLEU2 affected prostate cancer progression via the miR-582-5p/SGK1 axis.

### DLEU2 transcription was activated by aberrant expression of E2F2

**Fig. 6:**
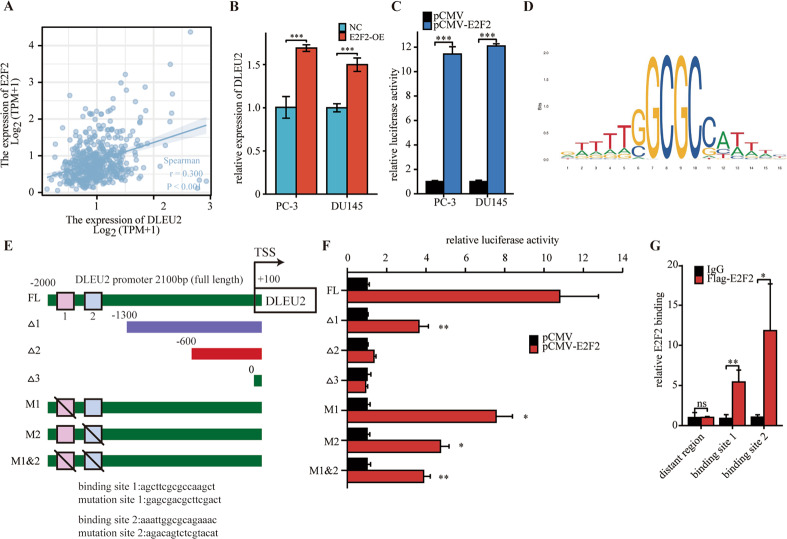
DLEU2 transcription was activated by aberrant expression of E2F2. **A** Correlation between E2F2 expression and DLEU2 expression in TCGA database using spearman analysis. **B** Relative expression of DLEU2 in PC-3 and DU145 cells transfected with E2F2 using RT-qPCR. **C** Relative luciferase reporter assays in PC-3 and DU145 cells after the co-transfection of plasmid constructs containing the DLEU2 promoter with a E2F2 overexpressing construct. **D** The DNA motif for E2F2 was obtained from JASPAR. **E** E2F2 binding sites in DLEU2 promoter were predicted by JASPAR. **F** Relative luciferase reporter assays in PC-3 cells after the co-transfection of a series of truncated and mutated DLEU2 promoter with a E2F2 overexpressing construct. **G** ChIP assay demonstrated the direct interactions between E2F2 and DLEU2 promoters in prostate cancer cells. Data were indicated as mean ± SD, ns *P* ≥ 0.05, *P < 0.05, **P < 0.01, ***P < 0.001.

We then investigated the cause of DLEU2 upregulation in advanced prostate cancer. Transcription factors are involved in cancer development. Thus, by analyzing TCGA database via GEPIA, we searched for transcription factors whose expression profile was correlated with DLEU2 expression. Importantly, we found that the expression of E2F2, which is known to be dysregulated in various cancers, was significantly related to DLEU2 expression (Fig. 6A). Therefore, we suspected that E2F2 may be involved in the regulation of DLEU2 expression. RT-qPCR results demonstrated that DLEU2 expression was upregulated in E2F2-overexpressing prostate cancer cells (Fig. 6B), and dual-luciferase reporter assays indicated that E2F2 transduction enhanced the transcriptional activity of the luciferase reporter flanked by the DLEU2 promoter in both PC-3 and DU145 cells, indicating that E2F2 may transactivate DLEU2 expression (Fig. 6C).

To further elucidate the related regulatory mechanisms, the promoter sequence of DLEU2 was analyzed using JASPAR (http://jaspar.genereg.net/), and two putative E2F2 binding motifs were found (Fig. 6D, E). Then, a series of luciferase reporter plasmids harboring truncated or mutated DLEU2 promoter sequences was constructed and transfected into PC-3 cells. The results indicated that −2000 bp to −1300 bp was essential for E2F2-induced expression of the luciferase reporter. Furthermore, site-directed mutagenesis of the DLEU2 promoter showed that binding sites 1 and 2 in the promoter were both indispensable for E2F2 binding (Fig. 6F). ChIP assays demonstrated that E2F2 directly bound to the DLEU2 promoter in PC-3 cells (Fig. 6G). Thus, our results showed that DLEU2 transcription was activated by aberrant E2F2 expression in prostate cancer.

### Knockdown of DLEU2 suppressed prostate tumor growth in vivo

**Fig. 7:**
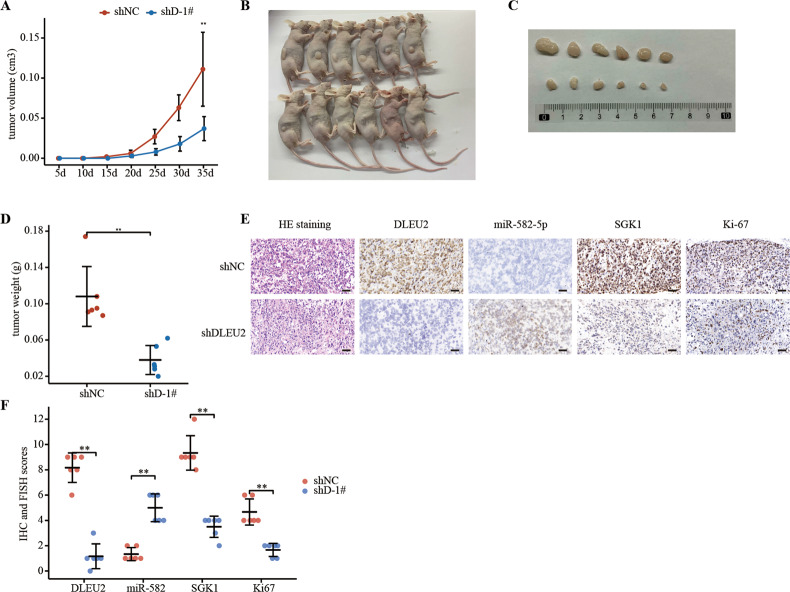
Knockdown of DLEU2 suppressed prostate tumor growth in vivo. **A** The tumor growth curve of xenografts was plotted in shNC and shDLEU2 group (*n* = 6 each group) by measuring the tumor size (0.5 × length × width^2^) each 5 days. **B** The subcutaneous tumor models were observed at 35 days in two different groups. **C** Images of xenograft tumors of each group (*n* = 6). **D** Weight of xenograft tumors of each group (*n* = 6). **E** Representative HE staining, IHC staining of Ki-67, SGK1 and FISH of DLEU2, miR-582-5p in tumor xenografts were conducted (scale bar: 50 μm). **F** The IHC scores of Ki-67, SGK1 and FISH scores of DLEU2, miR-582-5p in tumor xenografts. Data were indicated as mean ± SD, ns *P* ≥ 0.05, *P < 0.05, **P < 0.01, ***P < 0.001.

Using a xenograft mouse model, we found that sh-DLEU2 tumors grew slower than sh-Control tumors (Fig. 7A), yielding lower tumor volumes and weights (Fig. 7B–D). IHC and FISH analyses showed that DLEU2, Ki-67, and SGK1 were expressed at low levels in the DLEU2-knockdown group, whereas miR-582-5p was upregulated (Fig. 7E, F). Finally, hematoxylin and eosin staining (Fig. 7E) supported these findings. Overall, these data indicated that DLEU2 knockdown inhibited prostate carcinogenesis in vivo.

## Discussion

Prostate cancer is a common malignancy in men and is associated with high mortality rates [1]. The pathophysiological mechanisms driving prostate cancer progression are still unclear. Although PSA tests have been applied for clinical diagnosis for several decades, effective prognostic biomarkers have not been established. Accordingly, in this study, we utilized TCGA database and found that the lncRNA DLEU2 exhibited higher expression in advanced prostate cancer, consistent with previous findings in various other types of cancer. Moreover, consistent with recent studies in esophageal cancer and lung cancer, we found that high expression of DLEU2 was related to more advanced T stage and N stage and higher Gleason scores in prostate cancer and that DLEU2 upregulation was associated with poor survival rates. In addition, we showed that high DLEU2 expression was independently associated with a poor PFI. Overall, these data identified DLEU2 was a potential prognostic biomarker.

With the development of high-throughput sequencing technology, many lncRNAs have been shown to be critical for tumor progression. LncRNAs exert their roles at the transcriptional or post-transcriptional level to regulate the expression of downstream genes [35]. Importantly, lncRNAs are often differently expressed in tumor tissues and have been shown to participate in prostate cancer progression. For example, LINC00261 is upregulated in prostate cancer, and LINC00261 knockdown suppresses cancer cell viability and invasiveness [36]. Mechanistically, nuclear LINC00261 promotes the transcription of FOXA2, whereas cytoplasmic LINC00261 increases CBX2 expression by acting as an miR-8485 sponge [36]. In addition, PCAT1 contributes to the progression of prostate cancer by activating AKT and NF-κB signaling [14].

DLEU2 has been shown to play important roles in the progression of multiple cancers, including esophageal cancer, lung cancer, and hepatocellular cancer. Consistent with this, we found that DLEU2 was involved in prostate cancer proliferation, migration, and invasion. Furthermore, we found that DLEU2 overexpression promoted the proliferation, migration, and invasion of prostate cancer, whereas DLEU2 knockdown significantly inhibited prostate cancer progression. In vivo experiments indicated that DLEU2 knockdown suppressed prostate tumor growth. Thus, these results supported that DLEU2 acted as an oncogene in prostate cancer.

In this study, we identified SGK1 as a downstream of DLEU2; the results were confirmed in prostate cancer cells and in TCGA dataset, indicating that SGK1 expression was strongly suppressed by DLEU2 knockdown. We further showed that this mechanism involved miR-582-5p. miRNAs are a class of small noncoding RNAs approximately 22 nucleotides in length; these molecules are involved in regulating prostate cancer-related biological processes. For example, miR-146a is downregulated in androgen-independent prostate cancer tissues, and high expression of miR-146a induces apoptosis by inhibiting ROCK1 expression via targeting of the 3′ UTR [37]. Additionally, miR‐129‐5p promotes proliferation, migration, and invasion and blocks apoptosis in prostate cancer cells by regulating CAMK2N1 expression [38]. LncRNAs have been shown to regulate mRNA expression by acting as miRNA sponges [34]. Therefore, miRNAs are critical components of competing endogenous RNA (ceRNA) networks. Here, we demonstrated that DLEU2 regulated SGK1 expression by secluding miR-582-5p, which is involved in the pathogenesis of several types of cancer, including bladder cancer, osteosarcoma, and prostate cancer [39]. Indeed, low expression of miR-582-5p is positively correlated with advanced clinicopathological characteristics, whereas miR-582-5p overexpression inhibits invasion and migration by regulating transforming growth factor-β signaling. However, the mechanisms through which miR-582-5p is downregulated in prostate cancer are unclear. Our findings showed that miR-582-5p expression was regulated by DLEU2 and that DLEU2 regulated SGK1 expression by sponging miR-582-5p. Notably, inhibition of miR-582-5p partially rescued the inhibitory effects of DLEU2 on SGK1 expression in prostate cancer. Thus, we concluded that DLEU2 contributed to prostate cancer progression via the miR-582-5p/SGK1 axis.

Finally, we demonstrated that E2F2 contributed to DLEU2 overexpression in prostate cancer. Although many studies have described the functions of E2F2, the downstream genes of E2F2 in cancer have not been fully elucidated. Our data indicated that overexpression of E2F2 in prostate cancer cells contributed to upregulation of DLEU2, further resulting in aberrant regulation of the miR-582-5p/SGK1 axis.

**Fig. 8:**
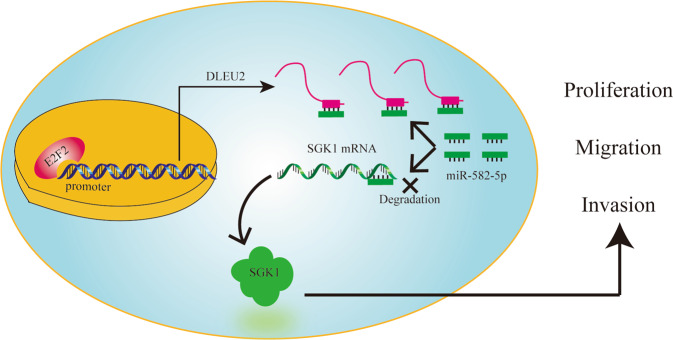
A schematic diagram for the role of DLEU2 in prostate cancer progression. E2F2 induces upregulation of DLEU2 by transcriptional activation. High expression of DLEU2 facilitates prostate cancer progression by acting as a miR-582-5p sponge to modulate SGK1 expression.

In summary, our findings demonstrated that DLEU2 overexpression in advanced prostate cancer tissues was correlated with poor outcomes. Moreover, we showed that DLEU2 acted as a regulator of cell proliferation, migration, and invasion. Mechanistically, E2F2-regulated DLEU2 acted as a ceRNA for miR-582-5p to modulate SGK1 expression (Fig. 8). Overall, these results suggested that DLEU2 may have applications as a biomarker for prostate cancer prognosis.

## Supplementary information

Reproducibility checklist

supplementary materials

Figure S1

Figure S2

Figure S3

Table S1

Table S2

## Data Availability

All remaining data are available within the article and supplementary files and are available from the authors upon request.
