# Supplementary material for: E2F transcription factor 2-activated DLEU2 contributes to prostate tumorigenesis by upregulating serum and glucocorticoid-induced protein kinase 1
Source: Cell Death Dis. 2022 Jan 24;13(1):77. doi: 10.1038/s41419-022-04525-1 (PMC8786838; doi:10.1038/s41419-022-04525-1)

**Supplementary Figure Legend:**

**Figure S1 CNCI score of DLEU2**

**Figure S2 DLEU2 was downregulated in advanced prostate cancer and was associated with a poor prognosis.**

**A** The correlation between T stage and miR-582-5p expression in prostate cancer. **B** The correlation between N stage and miR-582-5p expression. **C** The correlation between PSA levels and miR-582-5p expression. **D** The correlation between Gleason scores and miR-582-5p expression. **E** Kaplan Meier curves showed the correlation between miR-582-5p expression and PFI in patients with prostate cancer according to TCGA database.

**Figure S3 SGK1 expression**

Western blotting analysis of SGK1 expression in PC-3 and DU145 cells transfected with DLEU2 wild vectors or mutant vectors.

**Table S1: Sequences of the qRT-PCR primers used in this study**

**Table S2: Patients’ clinical information with different miR-582-5p expression level**

The detailed information of DLEU2 promoter sequence:

TTGCTTTCGCTCCCTCCTCTCCGCAGCTGGGAAGACTGCGCAAGAACAAAGTAGGGGCGAAAAGCGCGTGTCCTGACAGTGTGTCGGGGACACTGCAGCTTGGCGCGAAGCTAGGGCGGGGAAATGAAGGCTGAGGCCCGAGGCCTCTGTATCTCTGCCTCTCGCAGGTGTGGGACGGGCCAGGGGGCCCTGCCTTGTTTTCTTCATGCAAGGAAAAGTGGTTTGTAGATATTCAAAACAAAATTGGCGCAGAAACGAATTCGCCGTCTTAGGAGTGCTGTGGCAGGGCACAGCAGAAGCAGGAAGACGGGCGGCCAGGGCCTTTGCCTAAATTGGAAGGAAACCAGGAGCGAGCGCGCCAGCTCCCGCACCAGGCTCTGAGTACCGTTAGGTGTGAGGGGACCAAAGCGCCGTCCGGTTCGCTGGGGGGTCGACTAAGCTCCGCGTTTTGGAAGCCCAGCAAACACTGCACAGGTTCGCTTTACTTCCTCTCGCCTTCTTTAGAACGAGTGCACACATTGGCAGCTGACGATCCCACCACCTAGTCTCCCTCTTAAAAGAGGGCAGGCAAGCCTGGAAAGGTTTCCCAAATAGAGGCAAACTTATTGCTGTCTTGACTGGCCCAGCGAAAGCGCTCTCAGCACTGGCTGAGCCCAGTGCCCAGCCAGGAAGCTCGGCTCGGGTTGCGCGAGTCCTTGAAGAGTCCCCCACGGCCTGCAGTTGGCCCGAATGAGGGTCTGTGCGCGGGAACGGTTTCCCCAAACCCCTTAAGTAATAACATAACCCTTCCAGACTGTTCATCACACTGTTCAGCTGCCAGGAAAGGTGCGACGCGCTTCTTGCCCTCGTCCCAGCTCAGCTCTAGTTTCTTCCGCCAGTGAGCTGAGAAGCCAAATTATTTTATTCCCACCCTGTAAAGTTAAACTGCAATATACATAAATCACTCAGGGAAGCTGCTAAGATAAATAACACTCAAATGAGCTACTTGCACTTGGTATTGTTGTAAGGCCTTCCTCTGGTGTTTACCGCCCTCCTTGTTTACATGAAGCTCAGGAACAGCGGCTTTAGGCCGGTAGGGAGCTCAGTTTCATTTCAAGGGTATGGCAAGGCCTATCATTTCGTCATAAGGCTTTGAAGGAAAGTTCGTCTGTTTTTGTCTCACATCTAACTTCCAGTAACTTCTCAAAAACTATATTCTTCCAACTGTAGCTCCGGTCTGCAAACTCCCTTCTCCCTAGCTGTCCGGGAAGGGGGACTCGAGCTCGCGCAACGCCCCGCTGCACCTGCGGAGGAGGGAGAGCGTCTGGGTGCGCCATGGAACGCCCGCACCGCGCTTCCCGCGCTCCGCCCGCCTGCCCGCGAACGGCTGCGAGCCACCCCCGCATCCCTCACACCTCTGCCCTCCCCAGTCGTGCGCGGGAGAGGGGGCTAGCACCGCAATGCCTGGAAGAGCGGCCGCGCGAGGGGCCTGGGGACAGGACAGCCTCCCGGCTCCGAGGCGAGGCCCGAATGCGCGCACACTCGGCGAGCGGGGCGGCGACTGGGGAGCCGGAGACGAGAGAGGGCGGAAGTGCGGGACGCCCCGCAGCACTCCCACAGGTCGCGGAGCCCGAGATCCTCGTCGGGTGGCGAGGGCGTGGGTGCCCACGCTATGCGTGACGCCTGAGCCGCGGCCCGAGCCCACGAGCTGGGTGAGGCCGCTGCCGCCGCCGCCGCCACGAAGCCTGGCGGGCGGCGCGGAGCCCCGGGGAGCTGGTTTTCCCGGGCGGGCACGTGACGGGGTTGGCAGCGTTCGGGCCCCGGCTGGGAGGGGGAAGAAGCGGGGAGCAGAAGGCCGCGGGCGGGCGGCTGGCGCGCTGTGTACTTAGGTCGTGTGCTGGGGCTTTTCTCTCCCAGGAGCCGGCGGGGGGAGGGGAGGGGGAGGGGCCACCGCTCCGCCTTCTCCTTTTCGCAATGTTGACGCAATCTATAAATAGTGGAACAAAAGGACCAACTTCCTCCGTATTTTCCTTCTTCCCTGCATACCCCACAGAAGGAAAGCTGCTAAAATCTCAGAGCCGGAAAGTTCCAAGGGGGCTGCAGCAGCCCCTCTCTCCTAGT

The detailed sequence information of DLEU2 cloned into pMIRGLO

TAATTGATGCCAAAGAGTTCCAATATTGAACATCTTAAGTCTGTTACTTGGATTACGGATTGAGTTTGGAGCTTACTCAGAGGACTACAGGAGAGTATCCAGGAAGTGGATAATTACTGTACCTTCCTCATGGAAAAAGTTTTATTTAAAGTGTTATTTCTCATTGAATACTATCAAAAAGGAAAAAAAAATGACCTAAACTTTTGAGATAGATTTGGCTCTAGTAAGTATTTAGGCCTACTCTAGGTGCGGTACTTCACTATAGTTTATAAAAGATGGTCCCTGTCAGCAAAGAACTGTAACCTGTATGAGAATATTATACTAACATACAAGAAACAAGTAGAGAATAATGGAATGTAAACTCTGGCTTGCTAACTTAAGTGTGGTAGAAGCTTGAAGGAAATGTGGACTTAAACTTATCAGAGAAGACTTATAAGAGGATATGAAAGGTGTAAATTTTGATACCACCATTGCCGCCTTCCCCTCAAAAAAGGTGAGAACTGACTAAACTACTGGTACTTAGACCATGTGGAGAAGGAACTGAAAATGGAAACAGAAACACGTCTGTACAGAGCAGAGTGTCAGATAATAGCTCATTTT

The detailed sequence information of SGK1 cloned into pMIRGLO:

CTTTATTTTCCCTTGTGGATATGCTGTGTGAACCGTCGTGTGAGTGTGGTATGCCTGATCACAGATGGATTTTGTTATAAGCATCAATGTGACACTTGCAGGACACTACAACGTGGGACATTGTTTGTTTCTTCCATATTTGGAAGATAAATTTATGTGTAGACTTTTTTGTAAGATACGGTTAATAACTAAAATTTATTGAAATGGTCTTGCAATGACTCGTATTCAGATGCTTAAAGAAAGCATTGCTGCTACAAATATTTCTATTTTTAGAAAGGGTTTTTATGGACCAATGCCCCAGTTGTCAGTCAGAGCCGTTGGTGTTTTTCATTGTTTAAAATGTCACCTGTAAAATGGGCATTATTTATGTTTTTTTTTTTGCATTCCTGATAATTGTATGTATTGTATAAAGAACGTCTGTACATTGGGTTATAACACTAGTATATTTAAACTTACAGGCTTATTTGTAATGTAAACCACCATTTTAATGTACTGTAATTAACATGGTTATAATACGTACAATCCTTCCCTCATCCCATCACACAACTTTTTTTGTGTGTGATAAACTGATTTTGGTTTGCAATAAAACCTTGAAAAATA

shNC: TTCTCCGAACGTGTCACGTAA

shRNA targeting DLEU2:

shDLEU2-1: GTAAGTTCTTACCTTCGATTT

shDLEU2-2: CGTCGCACAATAAAGAAACAA


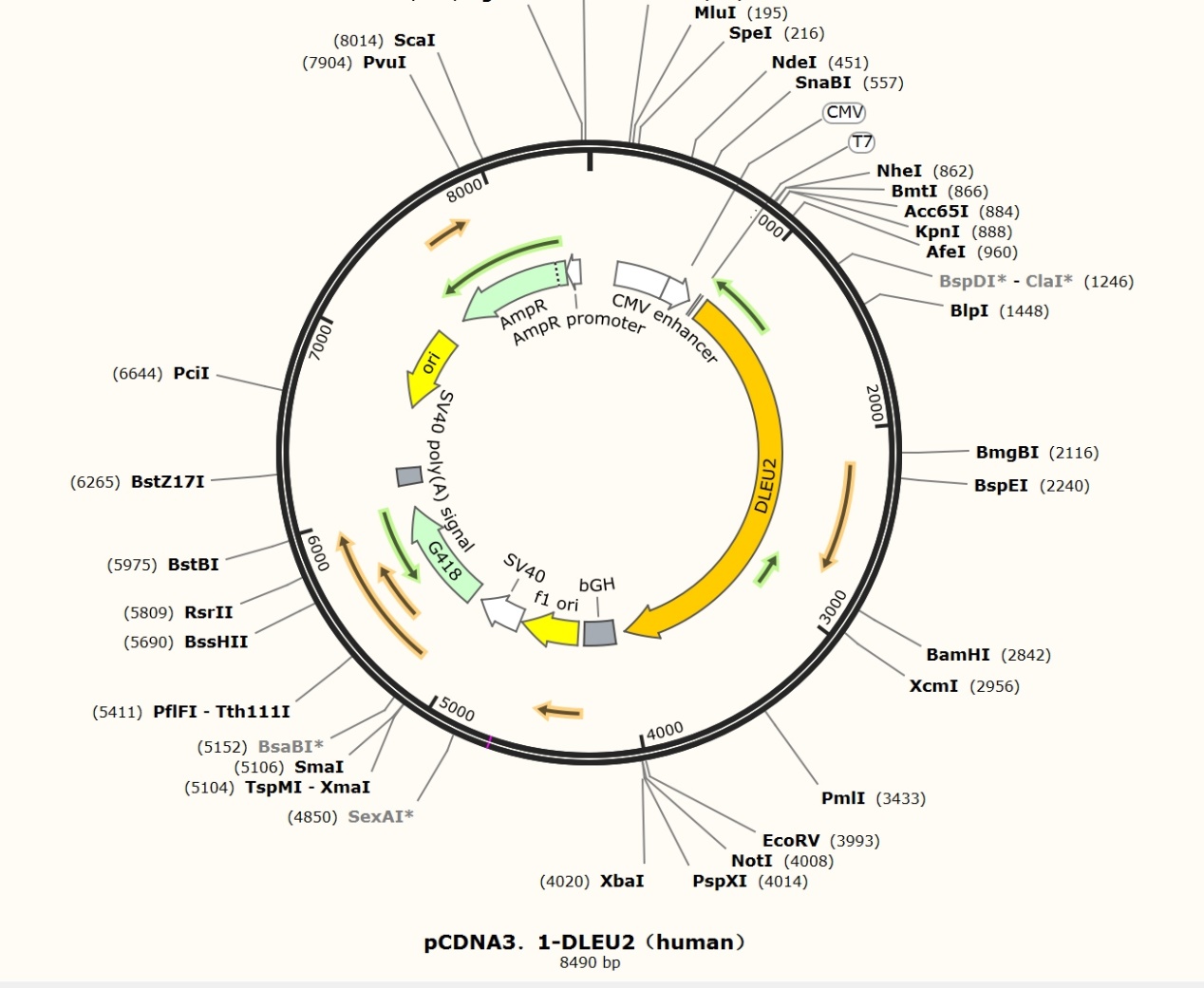


Fig3


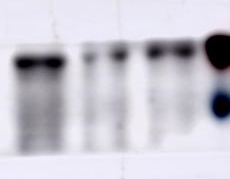


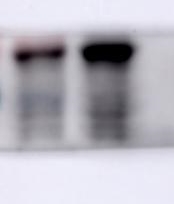


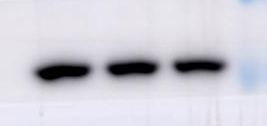

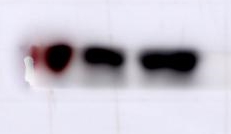


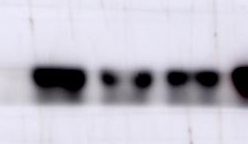


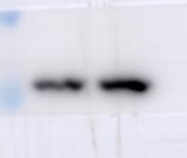


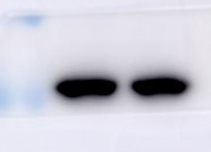

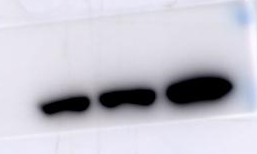


Fig4


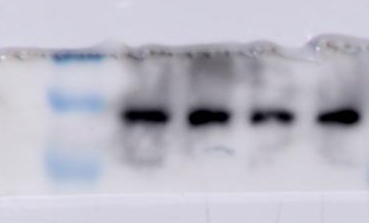

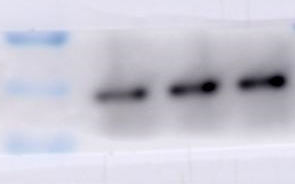

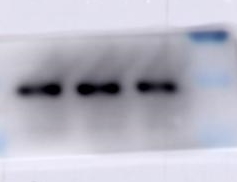

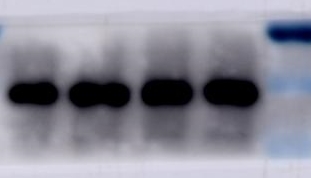


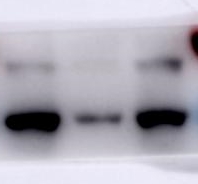

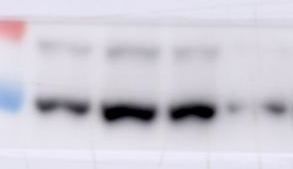

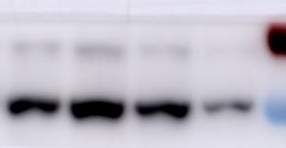

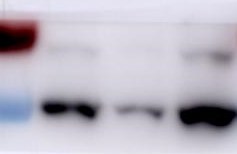


Fig5


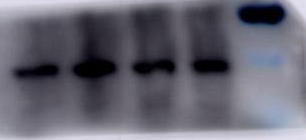

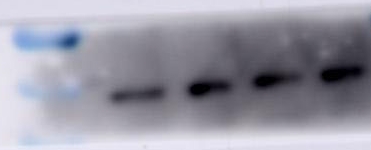


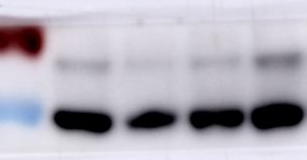

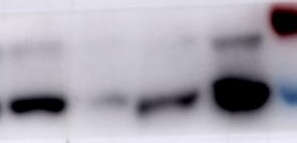

Supplement: Supplementary file 2 — supplementary materials [file 41419_2022_4525_MOESM2_ESM.docx]
