# Supplementary figures and images for: E2F transcription factor 2-activated DLEU2 contributes to prostate tumorigenesis by upregulating serum and glucocorticoid-induced protein kinase 1
Source: Cell Death Dis. 2022 Jan 24;13(1):77. doi: 10.1038/s41419-022-04525-1 (PMC8786838; doi:10.1038/s41419-022-04525-1)

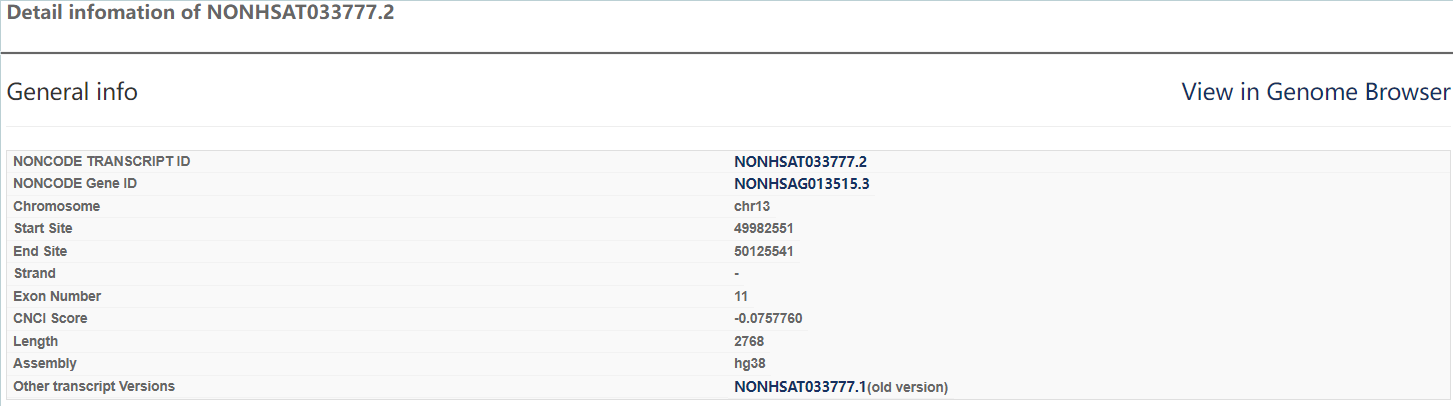

Supplement: Supplementary file 3 — Figure S1 [file 41419_2022_4525_MOESM3_ESM.png]

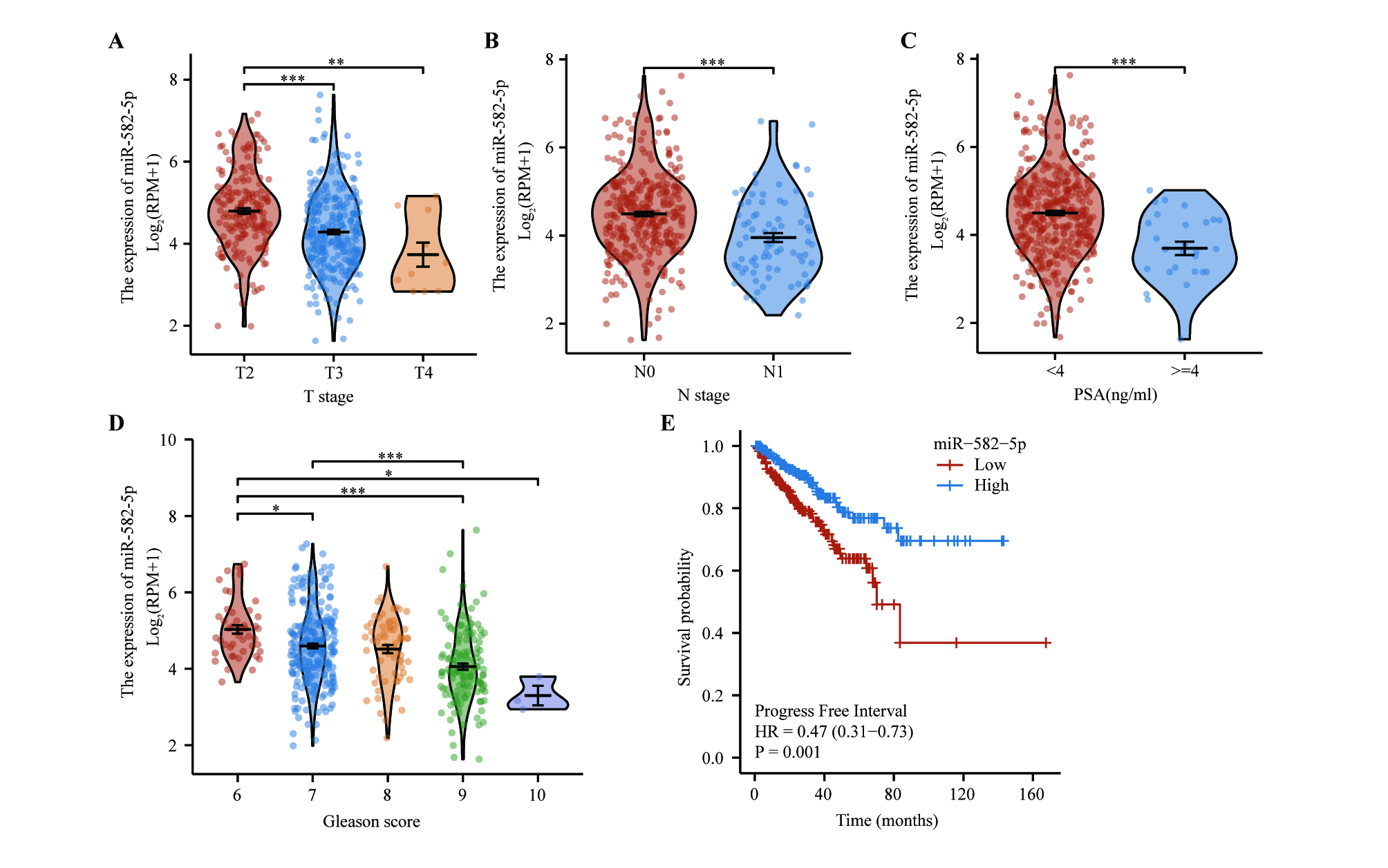

Supplement: Supplementary file 4 — Figure S2 [file 41419_2022_4525_MOESM4_ESM.tif]

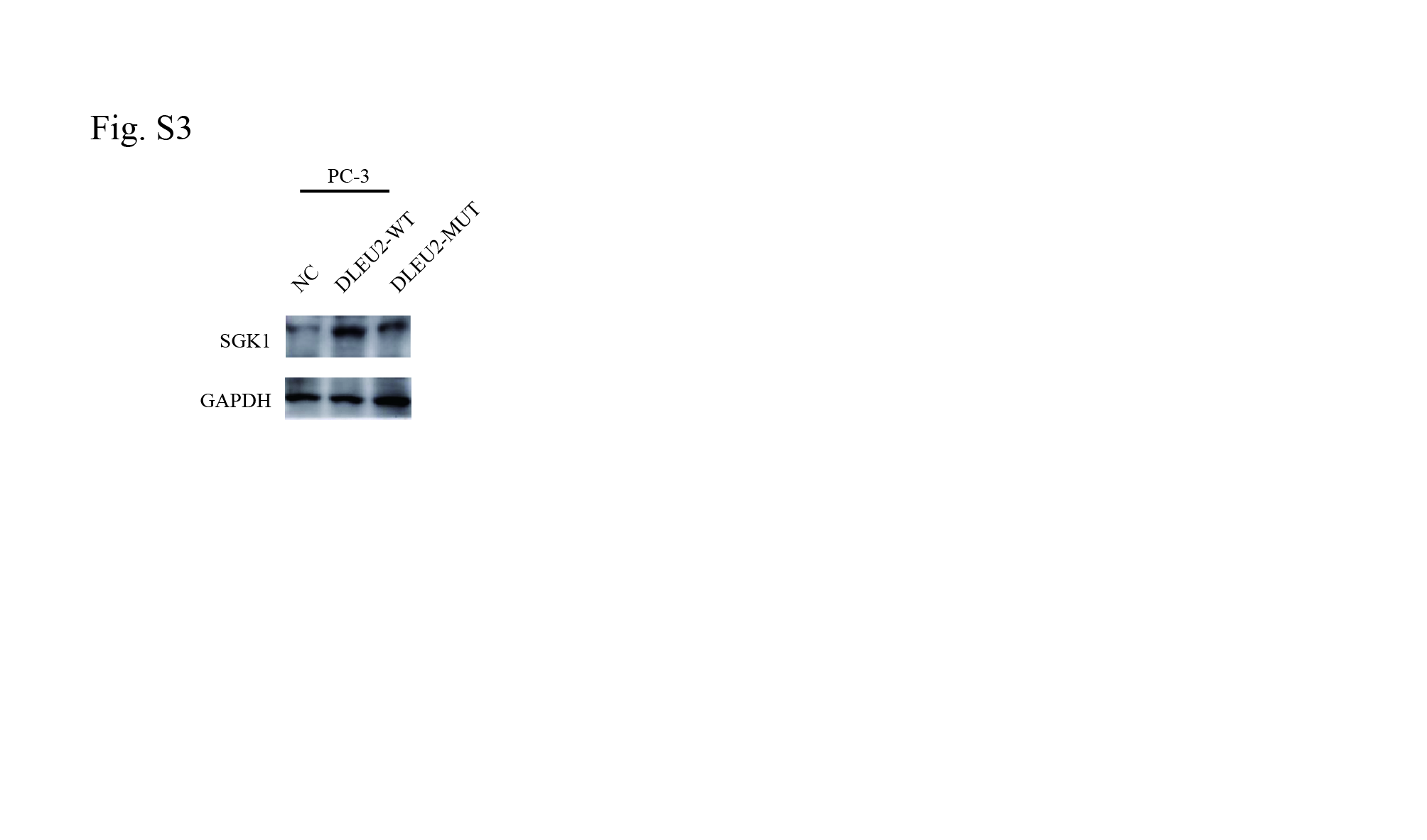

Supplement: Supplementary file 5 — Figure S3 [file 41419_2022_4525_MOESM5_ESM.tif]
