# Supplementary material for: E2F transcription factor 2-activated DLEU2 contributes to prostate tumorigenesis by upregulating serum and glucocorticoid-induced protein kinase 1
Source: Cell Death Dis. 2022 Jan 24;13(1):77. doi: 10.1038/s41419-022-04525-1 (PMC8786838; doi:10.1038/s41419-022-04525-1)
Supplement: Supplementary file 6 — Table S1 [file 41419_2022_4525_MOESM6_ESM.docx]

**Table S1: Sequences of the qRT-PCR primers used in this study**

| **Gene name** | **Forward primer (5’-3’)** | **Reverse primer (5’-3’)** |
| --- | --- | --- |
| DLEU2 | GCGGGTACTTATCTCCGACC | AGTGGCAGTTTCCCAGTCG |
| GAPDH | CTGGGCTACACTGAGCACC | AAGTGGTCGTTGAGGGCAATG |
| SGK1 | GCAGAAGAAGTGTTCTATGCAGT | CCGCTCCGACATAATATGCTT |
| miR-582-5p | AAGCGACCTTACAGTTGTTCAAC | GTCGTATCCAGTGCAGGGT |
| U6 | CTCGCTTCGGCAGCACA | AACGCTTCACGAATTTGCGT |
| Distant region | GCAAGCCTGATGGTGAGTCT | GTCATCAGAGGATGACGAGGT |
| Region 1 | GGGAAGACTGCGCAAGAACAA | CCTTCATTTCCCCGCCCTA |
| Region 2 | CCCTGCCTTGTTTTCTTCATGC | GGTCCGGTTTTCTGTCTGCT |
