# Supplementary material for: E2F transcription factor 2-activated DLEU2 contributes to prostate tumorigenesis by upregulating serum and glucocorticoid-induced protein kinase 1
Source: Cell Death Dis. 2022 Jan 24;13(1):77. doi: 10.1038/s41419-022-04525-1 (PMC8786838; doi:10.1038/s41419-022-04525-1)
Supplement: Supplementary file 7 — Table S2 [file 41419_2022_4525_MOESM7_ESM.docx]

**Table S2: Patients’ clinical information with different miR-582-5p expression level**

| Characteristic | Low expression of hsa-miR-582-5p | High expression of hsa-miR-582-5p | p |
| --- | --- | --- | --- |
| n | 249 | 250 |  |
| T stage, n (%) |  |  | < 0.001 |
| T2 | 64 (13%) | 126 (25.6%) |  |
| T3 | 173 (35.2%) | 119 (24.2%) |  |
| T4 | 7 (1.4%) | 3 (0.6%) |  |
| N stage, n (%) |  |  | 0.001 |
| N0 | 170 (39.9%) | 177 (41.5%) |  |
| N1 | 55 (12.9%) | 24 (5.6%) |  |
| M stage, n (%) |  |  | 0.249 |
| M0 | 230 (50.1%) | 226 (49.2%) |  |
| M1 | 3 (0.7%) | 0 (0%) |  |
| Age, n (%) |  |  | 0.034 |
| <=60 | 100 (20%) | 125 (25.1%) |  |
| >60 | 149 (29.9%) | 125 (25.1%) |  |
| PSA (ng/ml), n (%) |  |  | 0.002 |
| <4 | 203 (45.8%) | 213 (48.1%) |  |
| >=4 | 22 (5%) | 5 (1.1%) |  |
| Gleason score, n (%) |  |  | < 0.001 |
| 6 | 10 (2%) | 36 (7.2%) |  |
| 7 | 112 (22.4%) | 136 (27.3%) |  |
| 8 | 27 (5.4%) | 38 (7.6%) |  |
| 9 | 97 (19.4%) | 40 (8%) |  |
| 10 | 3 (0.6%) | 0 (0%) |  |
| PFI event, n (%) |  |  | 0.003 |
| Alive | 189 (37.9%) | 217 (43.5%) |  |
| Dead | 60 (12%) | 33 (6.6%) |  |
| Primary therapy outcome, n (%) |  |  | 0.012 |
| PD | 18 (4.1%) | 11 (2.5%) |  |
| SD | 21 (4.8%) | 9 (2%) |  |
| PR | 24 (5.5%) | 16 (3.6%) |  |
| CR | 155 (35.2%) | 186 (42.3%) |  |
| Age, meidan (IQR) | 62 (57, 66) | 60.5 (55, 65.75) | 0.010 |
